# Argon Laser Photoablation for Postburn Conjunctival Pigmentation

**DOI:** 10.1155/2014/586825

**Published:** 2014-11-18

**Authors:** Seong Joon Ahn, Jae Hoon Jeong, Hyo Jong Cho, Dong-Hoon Lee, Hyun Chul Kim

**Affiliations:** ^1^Department of Ophthalmology, Armed Forces Capital Hospital, No. 177, Saemaeul-ro, Bundang-gu, Seongnam, Gyeonggi-do 463-040, Republic of Korea; ^2^Department of General Surgery, Armed Forces Capital Hospital, No. 177, Saemaeul-ro, Bundang-gu, Seongnam, Gyeonggi-do 463-040, Republic of Korea

## Abstract

We report a case of an ocular burn injury from boiling water which resulted in conjunctival pigmentation, 1 week following injury. For cosmetic purposes, 2 sessions of argon laser photoablation were performed. One month after laser treatment, conjunctival pigmentation had been successfully removed and the patient was very satisfied with the results. Argon laser photoablation may be an effective way to remove postburn conjunctival pigmentation.

## 1. Introduction

Ocular chemical and thermal injuries represent between 8% and 18% of ocular traumas [1]. Necrosis of the conjunctival and corneal epithelium and loss of limbal stem cells occur and may result in persistent epithelial defects with sterile corneal ulceration and conjunctivalization/vascularization of the corneal surface leading to visual compromise [2, 3]. Thermal injuries can also cause aesthetic complications related to scarring and pigmentation [2]. Here, we report a case of postburn conjunctival pigmentation that was successfully removed with argon laser photoablation for cosmetic purposes.

## 2. Case Report

**Figure 1:**
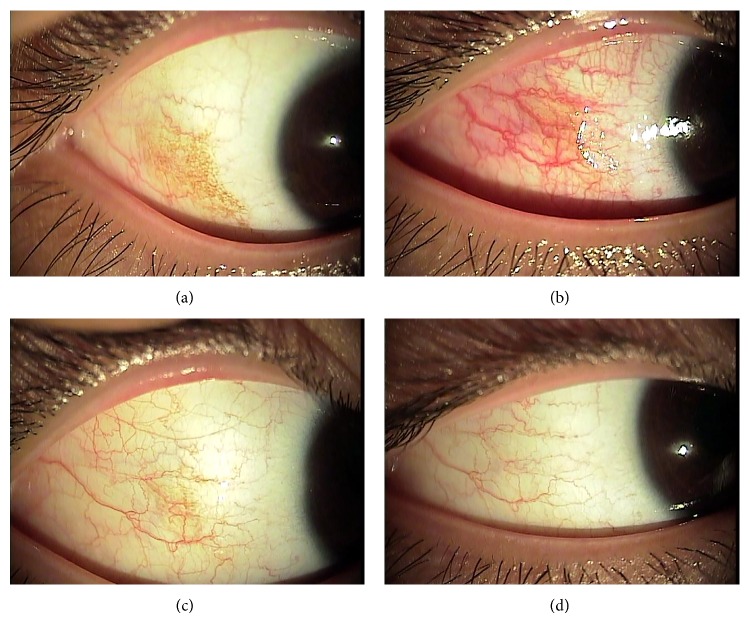
Anterior segment photographs obtained before and after the two argon laser photoablation sessions for postburn conjunctival pigmentation. (a) Pretreatment photograph showing conjunctival pigmentation on the temporal side of the right eye. (b) Immediately after the first argon laser photoablation session, approximately half of the pigmentation had been removed, leaving a conjunctival defect. Conjunctival injection around the laser-treated area is apparent. (c) One week after the first laser treatment, conjunctival injection had resolved and the remaining conjunctival pigmentation was removed with another argon laser photoablation session. (d) One month after the last treatment, no residual pigmentation, injection, or scarring of the temporal conjunctiva was present.

A 20-year-old man suffered from a thermal burn in the right eye and right periocular area from boiling water. The accident resulted in superficial burns on approximately 10% of the facial area. The patient received no treatment before coming to the hospital, 3 h after burning himself, where he was immediately sent to the emergency department. Physical examination revealed redness over the temporal periocular skin, and the patient reported mild pain over the burn area. Ocular examination showed punctate epithelial erosion and injection in the temporal interpalpebral conjunctiva of the right eye. The patient was given 0.5% levofloxacin (Cravit; Santen Pharmaceutical Company, Osaka, Japan), 0.1% fluorometholone acetate (Flarex; Alcon Laboratories, Fort Worth, TX), and 0.1% sodium hyaluronate (Kynex; Alcon Korea, Seoul, Korea) 4 times a day for 2 weeks. The patient returned to our clinic for a follow-up examination the next day. The skin in the right periocular area had developed a brownish pigmentation, which was subsequently removed as the eschar was peeled a few days later. One week after the thermal burn-inducing accident, the patient complained of a newly developed brown pigmentation in the temporal conjunctiva of his right eye. Neither the patient nor the examining physician had noticed or observed the pigmentation when the thermal burn occurred. Slit-lamp examination revealed a superficial conjunctival pigmentation, with a horizontal diameter of 7.8 mm and a vertical diameter of 5.5 mm, in the right eye (Figure 1(a)). All punctate epithelial erosions in the conjunctiva had completely healed.

Ten days later, conjunctival pigmentation persisted without any discernable change and the patient wished to have the cosmetically disturbing lesion removed. After discussing all treatment options (i.e., surgical excision and argon laser photoablation) with the patient, he chose to undergo argon laser therapy.

Two photoablation sessions were needed because of the large size of the pigmentation. These were performed in the right eye one week apart. After topical anesthesia was obtained by instilling a few drops of proparacaine hydrochloride 0.5% (Alcaine; Alcon Laboratories, FortWorth, TX), the laser was focused directly on an area of pigmentation. Laser spots were 200 *μ*m in size and did not overlap. The laser pulse had duration of 0.1 s and the energy delivered was 180 mW. In total, 140 and 124 laser spots were applied during the first and second laser sessions, respectively. The superficial conjunctival pigmentation was removed after argon laser photoablation followed by cotton-tipped swab massage of the laser-treated area. The patient had mild ocular pain after the laser photocoagulation, which lasted for 1 day. Immediately after the therapy, conjunctival injection was noted over the treated area (Figure 1(b)) and a conjunctival epithelial defect was left over the entire area of the treatment. For the epithelial defect, levofloxacin 0.5% ophthalmic solution (Cravit), and 0.1% fluorometholone acetate (Flarex) 4 times a day for one week were prescribed.

One week after the initial laser treatment, approximately half of the conjunctival pigmented area had been removed (Figure 1(c)). The treated eye completely healed, with no sign of inflammation, hemorrhage, or scarring. The second laser photoablation session was also uneventfully performed on any remaining pigmentation. One month after the second laser session, the temporal conjunctiva in the right eye had no residual pigmentation, injection, or scarring (Figure 1(d)) and the patient was very satisfied with the excellent cosmetic result. At the visit 6 months after the treatment, recurred pigmentation or scarring was not noted.

## 3. Discussion

Changes in skin pigmentation following a burn have been documented in many reports, but, to the best of our knowledge, postburn conjunctival pigmentation has not been reported in the literature. The mechanism by which conjunctival pigmentation forms following thermal injury is thought to be conjunctival melanosis. Inflammatory mediators released after a thermal burn occurs (e.g., leukotrienes and prostaglandins) may stimulate conjunctival melanocytes to increase melanin production [4]. Because the melanogenetic capacity of melanocytes correlates with the degree of ocular pigmentation (iris color) [5], it was more likely for our patient, who had dark brown eyes, to develop postburn conjunctival pigmentation compared to a patient with lighter colored eyes.

The goal of ocular surface burn therapy is to restore the ocular surface to normal and the cornea to clarity. Cosmetic concerns rarely play a role in therapy decisions. However, because of the white color of the conjunctiva and the visibility of the external ocular surface, the appearance of conjunctival pigmentation can be cosmetically upsetting to patients. Therefore, patients and physicians may decide to treat this condition, as was done in our case, even though the conjunctival and corneal surface has completely healed.

In a recent report by Shin et al. [6], argon laser photoablation effectively removed a superficial conjunctival nevus, a benign conjunctival pigmentation. The authors suggested that this method is especially helpful for removing large conjunctival nevi, for which surgical excision may result in scarring, dragging, and/or neovascularization [6]. In our patient with relatively large, amorphous conjunctival pigmentation, surgical excision could have left a large conjunctival defect and suturing the conjunctiva would have been technically difficult because of the pigmentation shape. Additionally, argon laser photoablation provided fine control of pigmentation removal, minimizing damage to surrounding normal tissues [7].

Our patient required two sessions of photoablation treatment, which may be concerning to some physicians. In our experience, a large number of laser burns (i.e., >100 burns) induce conjunctival injection, as shown in Figure 1(b). This made it difficult to distinguish between conjunctival pigmentation and normal conjunctiva. Also, one session of laser photoablation for large conjunctival pigmentation removal may leave a large conjunctival defect and induce severe pain both during and after the treatment. In our case, ocular discomfort increased in severity during laser therapy, so breaking the treatment up into two sessions was more tolerable for our patient.

To the best of our knowledge, this is the first report of conjunctival pigmentation caused by a thermal burn that was successfully removed with argon laser photoablation. The technique seems safe and effective for treating postburn conjunctival pigmentation, but the use of this technique needs to be verified in a larger number of cases.
